# The associations between problematic smartphone use and blood pressure among 2,573 aged 9–17 years students in Shanghai, China

**DOI:** 10.3389/fpubh.2022.904509

**Published:** 2022-09-07

**Authors:** Shaojie Liu, Yukun Lan, Gengsheng He, Bo Chen, Yingnan Jia

**Affiliations:** ^1^Department of Nutrition and Food Hygiene, School of Public Health, Fudan University, Shanghai, China; ^2^Department of Preventive Medicine and Health Education, School of Public Health, Fudan University, Shanghai, China

**Keywords:** blood pressure, problematic smartphone use, health behavior, students, cross-sectional study

## Abstract

**Objectives:**

This study aimed to (1) examine the cross-sectional association between problematic smartphone use (PSU) and blood pressure (BP) in children and adolescents and (2) determine whether the association between PSU and BP differs by the grade of students.

**Methods:**

We recruited a total of 2,573 participants from 14 schools in Shanghai by a two-stage sampling method. We derived BP data from the participant's most recent medical examination data, including systolic blood pressure (SBP) and diastolic blood pressure (DBP). We measured PSU by a modified PSU Classification Scale. We also assessed demographic characteristics, body mass index, behavioral variables, and physiological characteristics.

**Results:**

High school students had higher BP and PSU scores than primary and middle school students. PSU on the dimension of information collection was associated positively with both SBP and DBP among primary and middle school students, with the β (95% CI) values of 0.282 (0.018, 0.546) and 0.229 (0.031, 0.427). Meanwhile, the dimension of the relationship of social network was associated positively with SBP among primary and middle school students, with a β (95% CI) value of 0.390 (0.062, 0.717). PSU on the dimension of information collection was positively associated with the development of high BP, with a OR (95% CI) value of 1.072 (1.011, 1.137) among primary and middle school students.

**Conclusions:**

PSU might be a potential correlate of high BP among school-age children and adolescents. The associations between PSU and BP differed by the grade of students as well as according to the four dimensions of PSU.

## Introduction

As a public health concern, the prevalence of elevated blood pressure (BP) among children and adolescents has been increasing worldwide (1). Data from China Health and Nutrition Surveys 1991–2015 have suggested upward trends for 6–18 years participants in both systolic blood pressure (SBP) and diastolic blood pressure (DBP) levels, with significant increases in SBP of 17.2 mmHg and DBP of 11.6 mmHg in the average follow-up year of 7.3 years (2). The prevalence of hypertension among Chinese children and adolescents increased from 6.2% in 1991 to 14.1% in 2015, in concert with the prevalence of the unhealthy lifestyle and obesity in children and adolescents (3, 4). Elevated BP in children and adolescents is associated with an increased risk of early development of coronary artery disease and left ventricular hypertrophy as well as target organ damage (5, 6). Additionally, evidence indicates that hypertension tracks from childhood to adulthood, and adolescent hypertension has been linked to an increased risk of chronic disease in adulthood (7, 8). Although the upward trend in adolescent BP has been attributed largely to the increasing prevalence of obesity, other potential correlates, such as problematic smartphone use (PSU) in the context of advanced information and communication technology, also have contributed to this trend (9, 10).

Since the 1990s, studies on Internet and mobile phone use have been attracting increasing attention (11, 12). Dependence on Internet use refers to surfing the Internet using personal computers, and dependence on mobile phone use refers to time making phone calls and sending short message (13). Given the popularity of smartphones and the development of mobile Internet access, PSU has been proposed as a kind of dependent behavior (14).

School-age children and adolescents might be more susceptible to the adverse effects of smart media than older age groups (15), because they are highly receptive to new forms of media, and have less self-control ability than adults for smartphone (16). Physical and psychological problems among adolescents reportedly may result from PSU, including poor sleep quality (14, 17) as well as personality and psychiatric problems (15, 18, 19). Existing evidence has suggested that PSU was associated with several correlates of elevated BP, including physical activity (20), sedentary behavior (21), obesity (22), and depression (23, 24). To the best of our knowledge, however, there is no study investigating the direct relationship between elevated BP and PSU among children and adolescents, simultaneously.

Furthermore, previous measurements on PSU mainly were based on diagnostic criteria of substance addiction, including tolerance, compulsivity, withdrawal, and severe interference with daily life (25–27), which may lead to the inconsistent results for health effects (28). Many researchers, however, have suggested that whether it is dependence on Internet or smartphone use, computers and smartphones themselves are only tools, whereas their functions and content are the keys to understanding this dependence (29–31). Therefore, it is necessary to conduct research on PSU according to the various dimensions of content, such as the relationship of social network and entertainment.

Based on the function and content of smartphone use, we aimed to (1) examine the association between PSU and BP in 2573 children and adolescents and (2) determine whether this association between PSU and BP differs by the grade of students.

## Methods

### Informed consent

We obtained written informed consent statements from participants and also explained the right to withdraw and autonomy of the responses. The Ethics Committee of School of Public Health of Fudan University, China, approved this study (IRB2018120723 and FWA0002399).

### Questionnaire survey and physical measurement

We used a two-stage sampling method. First, we recruited a total of 14 schools in Shanghai based on the principle of voluntary participation and available medical examination data. School selection criteria include: (1) the principle of voluntary participation; (2) physical examination data available; (3) widely distributed in 15 districts of Shanghai, China; (4) half of the schools came from the suburbs and half from the city. Second, we randomly sampled at least two classes from each school with numbers of participants ranging from 100 to 250. Among the 14 schools, 5 were primary and middle schools, and 9 were high schools. The distribution of the participants in different grades and districts is shown in Supplementary Table 1. From December 14 to 27, 2017, 12 trained interviewers were responsible for collection and quality control of the self-administered questionnaires from each school in Shanghai. Teachers were enrolled to distribute the questionnaires. The detailed questionnaire design has been mentioned in previous study (32). All participants were asked to fill out a questionnaire regarding several variables, covering demographic information (e.g., age, sex, grade group), behavioral variables (indoor physical activity time, outdoor physical activity time, sedentary time, and screen time), and physiological characteristics (study pressure and well-being). Participants were required to wear light clothes and to stand straight on bare feet for height and weight measurements. We calculated body mass index (BMI) according to the weight divided by height squared (kg/m^2^). Of the 2625 students who were administered the survey, 2573 (98.0%) completed the survey for analysis.

### Variables explanation of questionnaire

The variables that indoor and outdoor physical activity time were evaluated by asking the questions “How long did you do indoor/outdoor physical activity per day in the recent school term? (including running, swimming, playing ball games, et al.)”. The variables that sedentary time and screen time were evaluated by asking the questions “What was your average sedentary time per day? (including class hours at school, homework at home, watching TV or playing games while sitting, et al.)” and “How many hours did you use electronic devices every day? (including mobile phone, computer, tablets, and other electronic devices with access to the Internet)”. Regarding the study pressure generally by a single survey question inviting students to provide a subjective assessment of the pressure they felt to study. On a five-point scale, participants answered the question, “Would you say that your study pressure is very much, much, moderate, a little, or none?” We measured well-being according to the Chinese version of the World Health Organization's Five Well-Being Index (33), which has demonstrated excellent psychometric properties in a large representative sample (34). Participants were asked to rate their status over the past 2 weeks by answering five questions, such as “How often have you felt active and vigorous?” Each item was rated on a 5-point scale ranging from 0 (never) to 5 (all the time). According to the total scores <13 points or ?13 points, the participants were divided into “poor well-being” or “good well-being,” respectively.

### Quality control

A number of quality-control measures were put in place to ensure the smooth running of the investigation. First, investigators can conduct this survey only after training for ensuring the quality of the investigation. Second, the investigators would answer any questions asked by participants to ensure that participants understood the content of the questionnaire, but did not interfere their responses. Third, the collected questionnaires were inputted and verified twice.

### Blood pressure data

We derived and checked BP data from the participant's most recent medical examination data from March, 2017 to December, 2017. In all 14 schools, we entrusted the doctors of community hospital to measure BP according to the recommendations by the National High Blood Pressure Education Program Working Group in Children and Adolescents using an auscultation mercury sphygmomanometer with an appropriate cuff size for students (35). In brief, after at least 5 min of rest, the operators obtained BP on the right arm of the seated participants with the elbow at the level of the right atrium. We defined SBP as the onset of the first Korotkoff sound and defined DBP as the fifth Korotkoff sound. The measurements were repeated twice. We calculated the mean of SBP and DBP measurements for each participant. We defined normal BP, pre-high BP, high BP stage 1, and high BP stage 2 according to the latest international BP references by sex, age, and height in children and adolescents 6–17 years old (36). Based on sex, age, and height of children and adolescents in this reference, BP percentile values could be used to define the different BP groups. Normal BP group was defined as SBP/DBP <90th percentile; pre-high BP group was defined as SBP/DBP ≥90th percentile and <95th percentile; high BP stage 1 group was defined as SBP/DBP ≥95th percentile and <99th percentile plus 5 mmHg; high BP stage 2 group was defined as SBP/DBP ≥99th percentile plus 5 mmHg.

### PSU measurement

On the basis of the previous study on the development of Smartphone Dependence Classification Scale (SDCS) for college students (37), we developed a revised scale according to the characteristics of urban adolescents in China. We investigated a convenient sample of 540 students from three schools in three grades. Results showed good reliability and validity. The Cronbach α of the whole scale was 0.828, and the Cronbach α for each subscale ranged from 0.600 to 0.800. The test–retest Intraclass Correlation Coefficient (ICC) of the whole scale was 0.736 (*P* < 0.01). The scale was composed of 15 items, which we categorized according to four dimensions: relationship of social network (four items), entertainment (five items), compulsive behavior (three items), and information collection (three items). The 15 Likert-scale items are presented in Supplementary Table 2. Each item ranged from 1 (strongly disagree) to 5 (strongly agree), in which a higher score indicated more serious PSU. We calculated the total score of each dimension, which we then included in the subsequent analysis as a continuous variable.

### Statistical analysis

We classified participants into two groups according to the grade of students: primary and middle school students, and high school students. On the one hand, many schools in Shanghai, China, are nine-year schools, including primary schools and middle schools. To ensure the understand of questionnaire among primary school students and reduce the interference other than academic work among middle school students, we mainly selected primary school students in grades 5–6 and middle school students in grades 7–8 as the research subjects. These subjects had a narrow age range. On the other hand, the sample size is relatively small in primary and middle school students. The numbers of primary and middle school students are of 555 and 319 participants, respectively. While the number of high school students is 1,699 participants. Thus, we merged primary and middle school students into a group for reducing the result bias. Continuous variables were described as median (inter-quartile range, IQR), including age, BMI, screen time, and study pressure, and categorical variables were described as frequency (ratio), including gender, indoor physical activity time, outdoor physical activity time, sedentary time, and well-being. The Chi-squared tests, and Wilcoxon rank-sum test were used for the equilibrium test in BP, PSU, and potential correlates of high BP between these two groups. The correlation analysis was used to examine the associations between PSU and BP among students from different grades. PSU was used as independent variables, and BP as the dependent variable. The multiple linear regression was used to examine the associations between PSU and BP of different grades adjusted for gender, age, BMI, indoor physical activity time, outdoor physical activity time, sedentary time, screen time, study pressure, and well-being. The interactions between PSU and grades on DBP and SBP were also explored by the multiple linear regression. As the dependent variable in logistic regressions, the variable was dichotomized as BP abnormality (high BP stage 1 and high BP stage 2) and BP normality (normal BP and pre-high BP). We conducted the multivariable logistic regression to examine the associations between PSU and BP of different grades adjusted for gender, age, BMI, indoor physical activity time, outdoor physical activity time, sedentary time, screen time, study pressure, and well-being. For all analyses, two-sided *P*-values <0.05 indicated statistical significance. Statistical analysis was performed using the Statistical Package for Social Sciences 20.0.

## Results

### Participant characteristics and potential correlates of BP

Table 1 shows the differences in demographic characteristics, health behaviors, physiological characteristics, PSU, and BP between the two groups. We identified significant differences in the distribution of gender, age, and BMI. Regarding health behaviors, primary and middle school students spent more time on physical activities than high school students and spent less time on sedentary behavior and using electronic devices than high school students. In terms of physiological characteristics, primary and middle school students felt significantly less study pressure and demonstrated better well-being compared with high school students. Whether we used BP as a continuous variable or as a categorical variable, high school students had a higher BP than primary and middle school students, especially in male students. Supplementary Table 3 displays that the personal characteristics of study participants based on the categories of PSU. Compared with primary and middle school students, high school students reported higher scores of PSU in all dimensions.

**Table 1:** Comparison of demographics characteristics, PSU, and potential correlates of blood pressure by grade of students [*N* (%)/Median (IQR)].

**Variables**		**Overall**	**Primary and middle school students**	**High school students**	***P*-value**
Gender	Male	1,221 (48.5)	448 (52.8)	773 (46.2)	0.002
	Female	1,299 (51.5)	400 (47.2)	899 (53.8)	
Age		16.0 (13.0, 17.0)	11.0 (10.0, 13.0)	16.0 (16.0, 17.0)	<0.001
BMI		20.3 (18.1, 23.0)	18.3 (16.5, 21.0)	21.0 (19.1, 23.7)	<0.001
Indoor physical activity time (min/d)	T <10	406 (15.9)	78 (9.1)	328 (19.4)	<0.001
	10 ≤ T <30	1,197 (47.0)	350 (40.9)	847 (50.1)	
	30 ≤ T <60	622 (24.4)	261 (30.5)	361 (21.3)	
	60 ≤ T <90	162 (6.4)	92 (10.8)	70 (4.1)	
	T ≥ 90	160 (6.3)	74 (8.7)	86 (5.1)	
Outdoor physical activity time (min/d)	T <10	72 (2.8)	29 (3.3)	43 (2.5)	<0.001
	10 ≤ T <30	524 (20.4)	108 (12.4)	416 (24.5)	
	30 ≤ T <60	1,139 (44.4)	333 (38.3)	806 (47.5)	
	60 ≤ T <90	555 (21.6)	264 (30.3)	291 (17.2)	
	T ≥ 90	276 (10.8)	136 (15.6)	140 (8.3)	
Sedentary time (h)	T <2	165 (6.4)	105 (12.1)	60 (3.5)	<0.001
	2 ≤ T <4	361 (14.1)	206 (23.7)	155 (9.1)	
	4 ≤ T <6	539 (21.0)	268 (30.8)	271 (16.0)	
	6 ≤ T <8	544 (21.2)	139 (16.0)	405 (23.9)	
	T ≥ 8	954 (37.2)	151 (17.4)	803 (47.4)	
Screen time (h)		2.0 (1.0, 3.0)	1.0 (0.5, 3.0)	2.0 (1.0, 3.5)	<0.001
Study pressure		3.0 (3.0, 4.0)	3.0 (2.0, 3.0)	3.0 (3.0, 4.0)	<0.001
Well-being	Poor	750 (29.4)	174 (20.2)	687 (38.1)	<0.001
	Good	1,805 (70.6)	687 (79.8)	1,118 (61.9)	
PSU on relationship of social network (out of 20 points)		8.0 (5.0, 11.0)	5.0 (4.0, 8.0)	9.0 (7.0, 12.0)	<0.001
PSU on entertainment (out of 25 points)		12.0 (7.0, 16.0)	8.0 (5.0, 13.0)	13.0 (9.0, 17.0)	<0.001
PSU on compulsive behavior (out of 15 points)		3.0 (3.0, 5.0)	3.0 (3.0, 3.5)	4.0 (3.0, 6.0)	<0.001
PSU on information collection (out of 15 points)		5.0 (3.0, 7.0)	5.0 (3.0, 7.0)	5.0 (3.0, 7.0)	<0.001
Total score of PSU		30.0 (22.0, 37.0)	23.0 (18.0, 31.0)	32.0 (26.0, 40.0)	<0.001
Boys	DBP SBP	117.0 (108.0, 127.0) 68.0 (62.0, 75.0)	110.0 (100.0, 118.0) 64.0 (60.0, 70.0)	120.0 (113.0, 130.0) 70.0 (63.0, 78.0)	<0.001 <0.001
Girls	DBP SBP	111.0 (104.0, 120.0) 70.0 (63.0, 74.0)	108.0 (100.0, 116.0) 66.0 (60.0, 70.0)	112.5 (105.0, 120.0) 70.0 (64.0, 76.0)	<0.001 <0.001
Stage of Hypertension among boys	Normal BP	652 (53.4)	311 (69.4)	341 (44.2)	<0.001
	Pre-high BP	247 (20.2)	52 (11.6)	195 (25.3)	
	High BP (Stage 1)	271 (22.2)	76 (17.0)	195 (25.3)	
	High BP (Stage 2)	50 (4.1)	9 (2.0)	41 (5.3)	
Stage of Hypertension among girls	Normal BP	874 (67.6)	279 (70.3)	595 (66.5)	0.126
	Pre-high BP	147 (11.4)	39 (9.8)	108 (12.1)	
	High BP (Stage 1)	225 (17.4)	71 (17.9)	154 (17.2)	
	High BP (Stage 2)	46 (3.7)	8 (2.0)	38 (4.2)	

IQR, inter-quartile range; BMI, body mass index; SBP, systolic blood pressure; DBP, diastolic blood pressure; BP, blood pressure; PSU, problematic smartphone use. There were some missing data existing in demographic characteristics of this study.

### Different associations between PSU and BP by the grade of students

We found that PSU was associated with BP in different grades of students by correlation analysis (Table 2). Among primary and middle school students, SBP was correlated positively with each dimension of PSU and total score of PSU (*P* < 0.05). Among high school students, however, SBP was correlated with PSU only on the entertainment dimension. Regarding DBP, except for the dimension of compulsive behavior, we identified significant positive correlations between DBP and the three other dimensions of PSU and total score of PSU among primary and middle school students (*P* < 0.05). In high school students, however, only the dimension of the relationship of social network was significantly associated with DBP.

**Table 2:** Differences of the associations between four separated dimensions of PSU and blood pressure by grade of students.

**Dimension of PSU**	**Systolic blood pressure**				**Diastolic blood pressure**			
	**Primary and middle school students**		**High school students**		**Primary and middle school students**		**High school students**	
	**Pearson coefficient**	***P***	**Pearson coefficient**	***P***	**Pearson coefficient**	***P***	**Pearson coefficient**	***P***
Relationship of social network	0.192	<0.001	−0.013	0.589	0.113	0.001	−0.056	0.023
Entertainment	0.112	0.001	0.052	0.032	0.103	0.003	−0.031	0.201
Compulsive behavior	0.090	0.008	−0.032	0.183	0.042	0.223	−0.031	0.209
Information collection	0.088	0.010	0.026	0.281	0.103	0.002	0.002	0.939
Total score of PSU	0.166	<0.001	0.023	0.342	0.136	<0.001	−0.040	0.106

PSU, problematic smartphone use.

Table 3 showed that the associations between PSU and BP by multiple linear regression analysis. After adjusting for gender, age, BMI, indoor physical activity time, outdoor physical activity time, sedentary time, screen time, study pressure, and well-being, the dimension of information collection was positively associated with SBP among the overall subjects, with a β (95% CI) value of 0.238 (0.044, 0.432); while the association was negative in the dimension of compulsive behavior. For the different grade groups, the dimension of information collection was associated positively with both SBP and DBP among primary and middle school students, with the β (95% CI) values of 0.282 (0.018, 0.546) and 0.229 (0.031, 0.427). Moreover, the dimension of the relationship of social network was associated positively with SBP among primary and middle school students, with a β (95% CI) value of 0.390 (0.062, 0.717). Among high school students, the dimension of compulsive behavior was negatively associated with SBP, with a β (95% CI) value of −0.509 (−0.850, −0.167). We further analyzed the interactions between PSU and grades on SBP and DBP (Supplementary Table 4). After adjusting for covariables, there was a negative interaction between the dimension of relationship of social network and grades on SBP and DBP, with β (95% CI) values of −0.344 (−0.679, −0.010) and −0.311 (−0.571, −0.052). Meanwhile, there was a negative interaction between total score of PSU and grades on DBP. with a β (95% CI) value of −0.117 (−0.205, −0.029).

**Table 3:** Associations between PSU and blood pressure by multiple linear regression analysis.

**Grade group**	**Dimension of PSU**	**Systolic blood pressure**		**Diastolic blood pressure**	
		**β (95% CI)**	***P***	**β (95% CI)**	***P***
Overall subjects	Relationship of social network	0.181 (−0.003, 0.365)	0.053	−0.104 (−0.247, 0.038)	0.152
	Entertainment	−0.036 (−0.161, 0.088)	0.569	0.033 (−0.063, 0.130)	0.500
	Compulsive behavior	−0.516 (−0.802, −0.229)	<0.001	−0.077 (−0.300, 0.145)	0.496
	Information collection	0.238 (0.044, 0.432)	0.016	0.137 (−0.014, 0.287)	0.075
	Total score of PSU	−0.003 (−0.059, 0.052)	0.905	−0.002 (−0.045, 0.041)	0.921
Primary and	Relationship of social network	0.390 (0.062, 0.717)	0.020	0.054 (−0.192, 0.300)	0.666
Middle school	Entertainment	−0.138 (−0.333, 0.057)	0.166	0.064 (−0.082, 0.211)	0.389
students	Compulsive behavior	−0.504 (-1.136, 0.128)	0.118	−0.328 (−0.803, 0.147)	0.175
	Information collection	0.282 (0.018, 0.546)	0.036	0.229 (0.031, 0.427)	0.023
	Total score of PSU	0.047 (−0.048, 0.142)	0.331	0.063 (−0.008, 0.134)	0.083
High school	Relationship of social network	0.169 (−0.053, 0.391)	0.136	−0.117 (−0.293, 0.060)	0.194
Students	Entertainment	−0.056 (−0.215, 0.103)	0.487	−0.024 (−0.150, 0.102)	0.708
	Compulsive behavior	−0.509 (−0.850, −0.167)	0.004	0.011 (−0.260, 0.283)	0.934
	Information collection	0.192 (−0.074, 0.458)	0.157	0.057 (−0.154, 0.269)	0.595
	Total score of PSU	−0.030 (−0.099, 0.038)	0.387	−0.030 (−0.084, 0.025)	0.286

PSU, problematic smartphone use. The models were adjusted for gender, age, BMI, indoor physical activity time, outdoor physical activity time, sedentary time, screen time, study pressure, and well-being.

Furthermore, we conducted multivariable logistic regression analysis to identify the associations between PSU and BP abnormality (Table 4). After adjusting for gender, age, BMI, indoor physical activity time, outdoor physical activity time, sedentary time, screen time, study pressure, and well-being, the dimension of information collection was positively associated with the development of high BP among the overall subjects, with a OR (95% CI) value of 1.057 (1.021, 1.095); while the association was negative in the dimension of compulsive behavior. For the different grade groups, the dimension of information collection was associated positively with the development of high BP, with a OR (95% CI) value of 1.072 (1.011, 1.137) among primary and middle school students. The dimension of compulsive behavior was associated negatively with the development of high BP, with a OR (95% CI) value of 0.915 (0.864, 0.969) among high school students.

**Table 4:** Associations between PSU and blood pressure abnormality by multivariable logistic regression analysis.

**Dimension of PSU**	**Model A**		**Model B**		**Model C**	
	**OR (95% CI)**	***P***	**OR (95% CI)**	***P***	**OR (95% CI)**	***P***
Relationship of social network	1.026 (0.993, 1.06)	0.121	1.051 (0.976, 1.132)	0.187	1.028 (0.990, 1.067)	0.153
Entertainment	0.984 (0.962, 1.006)	0.153	0.968 (0.925, 1.013)	0.156	0.985 (0.959, 1.012)	0.269
Compulsive behavior	0.915 (0.870, 0.963)	0.001	0.914 (0.790, 1.058)	0.231	0.915 (0.864, 0.969)	0.002
Information collection	1.057 (1.021, 1.095)	0.001	1.072 (1.011, 1.137)	0.021	1.044 (0.999, 1.091)	0.056
Total score of PSU	0.997 (0.987, 1.006)	0.482	1.005 (0.983, 1.026)	0.679	0.994 (0.983, 1.005)	0.282

PSU, problematic smartphone use. The model A for overall students; the model B for primary and middle school students; the model C for high school students. The model A, B, and C were adjusted for gender, age, BMI, indoor physical activity time, outdoor physical activity time, sedentary time, screen time, study pressure, and well-being.

## Discussion

To the best of our knowledge, this study was the first to examine the direct relationship between PSU and BP among primary, middle and high school students, which provides novel evidence. We found that there were significantly associations between PSU and BP especially for primary and middle students. Meanwhile, the associations between PSU and BP were different in the different dimensions of PSU and the different grades of student. There were the negative interactions between PSU and grades on DBP and SBP. In the future, we must employ a cohort study to further examine the causal relationship between PSU and BP among children and adolescents. In addition, the mechanisms related to PSU and BP remain to be clarified in future studies.

The present study suggested that the prevalence of pre-hypertension and hypertension differed by age and gender, which is consistent with some other studies (36, 38). Compared with another study of Germany children and adolescents with an average age of 11.9 years (39), the present study data from primary and middle school students with an average age of 11.4 years showed a higher average SBP (110.0 mmHg/109.2 mmHg) but a lower average DBP (64.7 mmHg/66.6 mmHg) both in boys and girls. For high school students with an average age of 16.3 years in the present study, average SBP and DBP were 121.7 and 71.1 mmHg in boys and 114.0 and 70.7 mmHg in girls, respectively, which were higher than the results from the study of Germany children and adolescents (39), except for boys' average DBP. Regarding the prevalence of pre-hypertension and hypertension, among high school students with the average age of 16.3 years, we found pre-hypertension and hypertension in 25.3 and 30.6% of the boys and in 12.1 and 21.7% of the girls, respectively. The prevalence of pre-hypertension and hypertension was higher in this study compared with several other countries, including in South Africa, where the prevalence of pre-hypertension and hypertension in 13–17-year-old adolescents was 13.6 and 22.0% in boys and 11.7 and 20.9% in girls, respectively (40). The prevalence of hypertension was higher among boys and lower among girls compared with a study in Suzhou, China, where the prevalence of hypertension in 13–18-year-old adolescents was 25.5% in boys and 24.2% in girls (41). This was attributed to the different races and dietary habits (42). Goulding et al. (43) found that elevated BP was most prevalent among US non-Latino Black children than those among US non-Latino White children based on the National Health and Nutrition Examination Survey 2011–2018 data. Meanwhile, Overwyk et al. (44) included 12,249 youths aged 8–17 years, and showed that the dietary habit of high sodium was positively associated with the elevated blood pressure/hypertension.

In this study, we used measurements on PSU primarily on functions and content of smartphone use rather than diagnostic criteria of substance addiction. Moreover, the revised PSU we developed did not have any grading criteria. Therefore, it was difficult to compare these results with previous studies (25–27). We did find, however, some distribution characteristics of PSU to be continuous variables. First, boys and girls had different distributions of types of PSU: except for the dimension of the relationship of social network, boys reported higher scores than girls in the other three dimensions. A study in China found similar results: girls had significantly higher scores on social network app dependence than boys (45). Another study in India revealed that boys were more likely to report mobile phone dependence than girls (46). Second, after standardization, the dimension of entertainment had the highest score among all four dimensions. A study among Chinese medical university students found that students with PSU used cell phones most frequently for music, whereas students without PSU used cell phones most frequently for social interaction (47). Last, the scores for PSU across all dimensions increased with the grade. A study in Italy, however, showed that the proportion of students who reported problematic cell phone use increased with the grade in girls but not in boys (48).

Regardless of whether we analyzed BP as a continuous variable or a categorical variable, our results from the correlation analyses showed that the relationship between PSU and BP was more significant in primary and middle school students than in high school students. On the one hand, this finding may indicate that the increased self-control ability of adolescents with increasing age may reduce the impact of PSU (15). On the other hand, parents and teachers play an important role in restricting smartphone use when adolescents enter the high school stage from the middle school stage. The majority of them considered that smartphone will delay the adolescents' study especially when high school students face severe academic competition, as with the notoriously difficult college entrance examinations (49), so that attenuated the association between PSU and BP among high school students. Moreover, compared with primary and middle school students, high school students had less exercise time, more sedentary time, and worse mental health as a result of the higher requirements for study (50, 51), which could weaken the relationship between PSU and BP. So far, the majority of literature only use screen time to explore the association between the use of electronic devices and adolescents' health (32, 52). However, increasing researchers argued that researchers should not only focus on screen time when exploring the health impact of the use of electronic devices for adolescents (53, 54). Huang et al. explored the associations of smartphone use with adolescents' social relationships in a large dataset, and found that their association relied on the various types of content during smartphone use (55). In this study, we also focused on the different dimensions of smartphone use to determine the association with BP. Thus, future studies are urgent to comprehensively assess the effect of smartphone use for health impact of adolescents, including screen time, screen type, content, and size.

Another interesting finding is that the correlation between the four dimensions of PSU and BP differed by the grade of students. After adjusting for other potential correlates, the dimensions of the relationship of social network and information collection were correlated positively with SBP and the development of high BP in primary and middle school students. The dimension of information collection and BP might have a positive association because higher scores for information collection might be associated positively with anger expression and distressed personality type, which have proven correlations with SBP (56, 57). For high school students, however, the dimension of compulsive behavior was found to be correlated negatively with SBP and the development of high BP. The dimension of compulsive behavior and BP might have a negative association because the scores for the dimension of compulsive behavior were relatively low among high school students, accounting for just 4.76 out of 15 points. The definition for this type of compulsive behavior was the traditional meaning of obsessive-compulsive disorder. According to the dimensions of PSU on compulsive behavior, students who reported higher scores of PSU on the dimension of compulsive behavior could have extroverted personalities. Previous studies have suggested that adolescents with extroverted personalities were less likely to have high BP compared with those who had introverted personalities (58, 59). Moreover, the association between personality characteristics and BP might be more significant in high school students than in primary and middle school students as a result of different pressure to study.

In general, our findings suggested that some dimensions of PSU might be associated with high BP among children and adolescents. Furthermore, the relationship may be related not only to the type of PSU but also to the grade of the student. The results were similar with the study of Zou et al. conducted in junior school students (60). They found the significantly positive association of smartphone addiction with hypertension, and there was an increasing trend with the increase of student grade (60). Not all PSU behaviors are associated positively with high BP. Some PSU behaviors, such as checking for and replying to message updates as soon as possible, might be associated negatively with high BP in the case of students with relatively low scores on PSU. However, the mechanisms related to PSU and BP remains to be clarified in future studies. Regarding the grade of students, some differences were revealed between PSU and BP according to the grade of students in both unadjusted and adjusted models. Especially for students from primary and middle schools who might be susceptible to the adverse effects of smart media (15), PSU on the dimensions of the relationship of social network and information collection might be related to high BP. Especially in the context of the global COVID-19 outbreak, the reduction in time spent outdoors may exacerbate PSU. Therefore, more targeted interventions on PSU need to be developed based on the student's grade and characteristics. For example, avoiding excessive information contact, including news and information from social apps, may reduce the risk of high BP, whereas it might be not bad for high school students to demonstrate some compulsive behaviors on PSU, such as checking for and replying to message updates in social apps. Moreover, considering the associations between PSU and several correlates of high BP (20–23), the strategies to prevent PSU should be considered in conjunction with prevention strategies for other risk factors of high BP, such as physical inactivity and sedentary behaviors.

This study has some limitations. First, the direction of causality could not be addressed because of the cross-sectional study design. It is unable to generalize the results to other regions because the overall subjects were from Shanghai, China. Second, the schools failed to be completely selected randomly even though some measures were taken to avoid bias, which may influence the results of this study. PSU has not been used widely, so it is difficult to compare these results with existing studies. Third, although we found some valuable results, powerful explanations were insufficient to explain the causes of the potential relationship between PSU and high BP based on the cross-sectional study. Meanwhile, we failed to distinguish the characteristics of the different screen devices in the questionnaire survey, such as screen time, screen type, content, and size, which would have different influences on children's health. Last, data were acquired from 2017, which may be unable to reflect the present status due to the rapid development of electronic technology.

## Conclusion

PSU might be a potential correlate of high BP among school-age children and adolescents. The association between PSU and BP differed by the grade of students as well as by the dimension of PSU. When considering other risk factors for high BP, it is necessary to develop targeted interventions on PSU based on the grade of students and other student characteristics.

## Data availability statement

The raw data supporting the conclusions of this article will be made available by the authors, without undue reservation.

## Ethics statement

The studies involving human participants were reviewed and approved by the Ethics Committee of School of Public Health of Fudan University, China, approved this study (IRB2018120723 and FWA0002399). Written informed consent to participate in this study was provided by the participants' legal guardian/next of kin.

## Author contributions

SL and YJ designed the research, wrote the paper, and had primary responsibility for the final content. YL provided the data. SL performed the statistical analyses. YJ, GH, and BC reviewed and edited the manuscript. All authors have read and agreed to the published version of the manuscript.

## Funding

The work was supported by the Shanghai Municipal Health Commission (Grant Numbers: GWV-10.2-YQ23 and GWV-10.1-XK14), and the USyd-Fudan Partnership Collaboration Awards. The funders had no role in study design, data collection and analysis, decision to publish, and preparation of the manuscript.

## Conflict of interest

The authors declare that the research was conducted in the absence of any commercial or financial relationships that could be construed as a potential conflict of interest.

## Publisher's note

All claims expressed in this article are solely those of the authors and do not necessarily represent those of their affiliated organizations, or those of the publisher, the editors and the reviewers. Any product that may be evaluated in this article, or claim that may be made by its manufacturer, is not guaranteed or endorsed by the publisher.
