# Supplementary material for: The associations between problematic smartphone use and blood pressure among 2,573 aged 9–17 years students in Shanghai, China
Source: Front Public Health. 2022 Sep 7;10:904509. doi: 10.3389/fpubh.2022.904509 (PMC9490019; doi:10.3389/fpubh.2022.904509)
Supplement: Supplementary file 1 [file Data_Sheet_1.pdf]

Table S1. Distribution of the participants in different grades and districts

Location	Number of participants		Total
	Primary and middle school	High school	
Urban_1	/	202	202
Urban_2	137	/	137
Urban_3	/	187	187
Urban_4	/	205	205
Urban_5	/	214	214
Urban_6	110	/	110
Urban_7	/	198	198
Suburban_1	/	103	103
Suburban_2	/	200	200
Suburban_3	/	176	176
Suburban_4	209	/	209
Suburban_5	/	214	214
Suburban_6	215	/	215
Suburban_7	203	/	203
Total	874	1699	2573

Table S2. Smartphone Dependence Classification Scale

Dimension	Item
Relationship of social network	1. I spend a lot of time on a daily basis on social apps (QQ, WeChat, Weibo, etc.).
	2. I usually have a late bedtime after using social apps in bed.
	3. I spend more and more time on social apps.

	4. Because of chatting on the Internet, parents and friends have opinions about me, but I still haven't reduced the time for online chat.
Entertainment	5. I enjoy using entertainment apps, e.g., playing mobile games, watching videos, reading novels, etc.
	6. Using entertainment apps is my favorite way to relax and reduce stress.
	7. I always keep up with updates from online novels and/or games.
	8. I spend a lot of time on mobile entertainment.
	9. I have a strong sense of achievement and satisfaction after completing a game, video series, or novel.
Compulsive behavior	10. I cannot resist the impulse to check for message updates in social apps (QQ, WeChat, Weibo, etc.).
	11. I always reply to the news on social apps, even sneaking back during class; otherwise, I will feel uneasy.
	12. Even thinking about using entertainment apps (gaming, video, novel, etc.) distracts me from other activities.
Information collection	13. I will read the news pushed by the mobile phone apps in the first time.
	14. I spend a lot of time on a daily basis searching for and reading various news and information.
	15. If I do not read news, I will feel restless.

Table S3. Personal characteristics of study participants based on the categories of PSU [Median (IQR)]

Characteristics		PSU on relationship of social network	PSU on entertainment	PSU on compulsive behavior	PSU on information collection	Total score of PSU
Gender	Male	8 (5, 11)	13 (9, 17)	3 (3, 5)	5 (3, 8)	31 (23.5, 39)
	Female	8 (5, 11)	10 (7, 14)	3 (3, 5)	5 (3, 7)	28 (21, 36)
Grade	Primary and middle school	5 (4, 8)	8 (5, 13)	3 (3, 3.5)	5 (3, 7)	23 (18, 31)
	High school	9 (7, 12)	13 (9, 17)	4 (3, 6)	5 (3, 7)	32 (26, 40)
SBP sort	Normal BP	8 (5, 11)	11 (7, 16)	3 (3, 5)	5 (3, 7)	29 (21, 36)
	Pre-high BP	9 (6, 12)	13 (9, 16)	3 (3, 5)	5 (3, 7)	32 (25, 38)

	High BP (Stage 1)	8 (5, 12)	12 (8, 16)	3 (3, 5)	5 (3, 8)	31 (24, 40)
	High BP (Stage 2)	8 (6, 12)	11.5 (8, 16)	3 (3, 5)	5 (3, 7)	29 (24, 37)
DBP sort	Normal BP	8 (5, 11)	12 (7, 16)	3 (3, 5)	5 (3, 7)	29 (22, 37)
	Pre-high BP	8 (6, 11.5)	12 (8, 15)	3 (3, 5)	5 (3, 7)	31 (25, 37)
	High BP (Stage 1)	8 (6, 11)	12 (9, 15)	3 (3, 5)	6 (4, 8)	31 (25, 37)
	High BP (Stage 2)	7 (5, 11)	11 (7, 17)	3 (3, 4)	5 (3, 8)	26 (23, 35)
Well-being	Poor	9 (6, 12)	13 (9, 17)	4 (3, 6)	5 (3, 7)	31 (25, 39)
	Good	8 (5, 11)	11 (7, 15)	3 (3, 5)	5 (3, 7)	29 (21, 36)
Indoor physical activity time (min/d)	T < 10	9 (6, 12)	13 (9, 17)	4 (3, 6)	5 (3, 7)	33 (25, 40)
	10 ≤ T < 30	8 (5, 12)	12 (8, 16)	3 (3, 5)	5 (3, 7)	31 (24, 38)
	30 ≤ T < 60	7 (4, 11)	10 (6, 15)	3 (3, 5)	5 (3, 7)	27 (19, 36)
	60 ≤ T < 90	6 (4, 9)	10 (6, 15)	3 (3, 4)	5 (3, 7)	26 (19, 34)
	T ≥ 90	6 (4, 10)	10 (6, 15.5)	3 (3, 4)	4.5 (3, 7)	26 (19, 34.5)
Outdoor physical activity time (min/d)	T < 10	11 (6, 16)	15 (8, 20)	4 (3, 9)	6 (4, 11)	36 (23, 50)
	10 ≤ T < 30	9 (6, 12)	12 (8, 16)	4 (3, 6)	5 (3, 8)	32 (24, 39)
	30 ≤ T < 60	8 (5, 12)	12 (8, 16)	3 (3, 5)	5 (3, 7)	30 (24, 37)
	60 ≤ T < 90	7 (4, 10)	10 (6, 15)	3 (3, 4)	5 (3, 7)	26 (19, 35)
	T ≥ 90	6 (4, 9)	10.5 (6, 15)	3 (3, 4)	5 (3, 7)	27 (20, 34)
Sedentary time (h)	T < 2	10 (7, 16)	15 (10, 20)	4 (3, 8)	6 (4, 8)	32 (27, 49)
	2 ≤ T < 4	9 (6, 12)	13 (9, 16)	4 (3, 6)	5 (3, 7)	31.5 (25, 39)
	4 ≤ T < 6	8 (5, 11)	12 (8, 16)	3 (3, 5)	5 (3, 7)	30 (23, 37)
	6 ≤ T < 8	6 (4, 10)	10 (5, 15)	3 (3, 4)	5 (3, 7)	26 (18, 36)
	T ≥ 8	6 (4, 10)	11 (7, 15)	3 (3, 4)	5 (3, 7)	27 (21, 35)

Abbreviation: IQR, Inter-quartile range; PSU, problematic smartphone use; BP, blood pressure; SBP, systolic blood pressure; DBP, diastolic blood pressure.

Table S4. The interactions between PSU and grades on blood pressure

Dimension of PSU	Systolic blood pressure		Diastolic blood pressure	
	β (95% CI)	*P*	β (95% CI)	*P*
Relationship of social network	0.681 (0.061, 1.300)	0.031	0.469 (-0.011, 0.949)	0.055
Relationship of social network*grades	-0.344 (-0.679, -0.010)	0.044	-0.311 (-0.571, -0.052)	0.019
Entertainment	-0.096 (-0.477, 0.284)	0.620	0.269 (-0.026, 0.564)	0.074
Entertainment*grades	0.041 (-0.174, 0.255)	0.712	-0.159 (-0.325, 0.008)	0.062
Compulsive behavior	0.118 (-1.155, 1.392)	0.855	0.072 (-0.916, 1.060)	0.886
Compulsive behavior*grades	-0.212 (-0.878, 0.454)	0.532	-0.086 (-0.603, 0.431)	0.744
Information collection	0.491 (-0.129, 1.111)	0.121	0.541 (0.061, 1.022)	0.027
Information collection*grades	-0.222 (-0.586, 0.141)	0.231	-0.267 (-0.549, 0.014)	0.063
Total score of PSU	0.115 (-0.093, 0.323)	0.278	0.201 (0.040, 0.362)	0.014
Total score of PSU*grades	-0.064 (-0.177, 0.050)	0.272	-0.117 (-0.205, -0.029)	0.010
